# Outcomes of endoscopic mucosal resection for large superficial non-ampullary duodenal adenomas

**DOI:** 10.1038/s41598-022-18528-7

**Published:** 2022-08-26

**Authors:** Maxime Amoyel, Arthur Belle, Marion Dhooge, Einas Abou Ali, Anna Pellat, Rachel Hallit, Benoit Terris, Frédéric Prat, Stanislas Chaussade, Romain Coriat, Maximilien Barret

**Affiliations:** 1 Gastroenterology Department, Cochin Hospital, Assistance Publique - Hôpitaux de Paris, 27 rue du Faubourg Saint Jacques, 75014 Paris, France; 2 Gastroenterology Department, Beaujon Hospital, Assistance Publique - Hôpitaux de Paris, Clichy, France; 3 Université de Paris Cité, Paris, France; 4 Pathology Department, Cochin Hospital, Assistance Publique - Hôpitaux de Paris, Paris, France

**Keywords:** Cancer prevention, Gastrointestinal cancer, Oesophagogastroscopy, Gastroenterology

## Abstract

Endoscopic mucosal resection (EMR) is the recommended treatment for superficial non-ampullary duodenal epithelial tumors larger than 6 mm. This endoscopic technique carries a high risk of adverse events. Our aim was to identify the risk factors for adverse events following EMR for non-ampullary duodenal adenomatous lesions. We retrospectively analyzed a prospectively collected database of consecutive endoscopic resections for duodenal lesions at a tertiary referral center for therapeutic endoscopy. We analyzed patients with non-ampullary duodenal adenomatous lesions ≥ 10 mm resected by EMR, and searched for factors associated with adverse events after EMR. 167 duodenal adenomatous lesions, with a median size of 25 (25–40) mm, were resected by EMR between January 2015 and December 2020. Adverse events occurred in 37/167 (22.2%) after endoscopic resection, with 29/167 (17.4%) delayed bleeding, 4/167 (2.4%) immediate perforation and 4/167 (2.4%) delayed perforation. In logistic regression, the size of the lesion was the only associated risk factor of adverse events (OR = 2.81, 95% CI [1.27; 6.47], p = 0.012). Adverse events increased mean hospitalization time (7.7 ± 9 vs. 1.9 ± 1 days, p < 0.01). None of the currently recommended preventive methods, particularly clips, affected the adverse event rate. EMR of centimetric and supracentimetric duodenal adenomatous lesions carries a high risk of adverse events, increasing with the size of the lesion and with no benefit from any preventive method. These results suggest that these procedures should be performed in expert centers, and underline the need for novel endoscopic tools to limit the rate of adverse events.

## Introduction

Superficial non-ampullary duodenal epithelial tumors (SNADETs) are encountered in 0.1–0.8% of upper gastrointestinal endoscopies^1–3^. They can be sporadic or occur in the setting of a hereditary predisposition syndrome, mainly familial adenomatous polyposis (FAP). Duodenal adenomas have the potential to progress towards invasive adenocarcinoma, warranting resection for any sporadic adenoma and for all significant lesions (> 10 mm or sign of high-grade dysplasia) in patients with FAP^4^. The progresses of endoscopic techniques allowing for a better detection of duodenal adenomas, explain the apparent increasing incidence of these lesions^3^. The specific anatomical conditions of the duodenum, such as the thin muscle layer, the narrow lumen and the rich vascularization, make the endoscopic treatment more challenging than in other parts of the gastrointestinal tract. In 2021, the European Society of Gastrointestinal Endoscopy (ESGE) released guidelines advising to practice cold snare polypectomy for lesions < 6 mm, endoscopic mucosal resection (EMR) being the first-line endoscopic resection technique for non-malignant SNADETs over 6 mm in size^5^. Studies reporting on the outcomes of EMR for duodenal adenomas, including large lesions, and endoscopic follow-up data on a significant number of patients are scarce. The aim of this study is to determine the rate and the risk factors of clinically significant adverse events (AE) following EMR of large duodenal adenomatous lesions.

## Patients and methods

### Patients

Consecutive patients who underwent endoscopic resection at our tertiary digestive endoscopy center from January 2015 to December 2020 were included. We included all patients treated by EMR for SNADETs ≥ 10 mm. We did not include patients with ampullary adenoma, non-adenomatous histology (neuroendocrine tumor, Brunner’s gland hyperplasia, gastric ectopic mucosa…), or treated by endoscopic submucosal dissection or by a hybrid resection technique.

This is a retrospective study of a prospectively collected database. Patients' clinical and endoscopic data were extracted from computerized medical records.

### Endoscopic procedure and follow up

All methods were carried out in accordance with relevant guidelines and regulations. Endoscopy was performed at a tertiary endoscopy center by five expert endoscopists under general anesthesia with orotracheal intubation, CO2 insufflation, and in the supine position. A gastroscope with or without a cap, a duodenoscope, a pediatric colonoscope, or a combination of different endoscopes were used for morphological characterization of the lesions and resection, according to location. Duodenal polyps were described according to the Paris classification^6^, and their size was assessed by the operator in the report, or if necessary by consultation of the endoscopy images. In accordance with Klein et al., lesions were classified as large (10–29 mm) or giant (≥ 30 mm)^7^. When lesion margins were not well defined, virtual chromoendoscopy (Narrow Band Imaging (NBI), Olympus, Japan; Fuji Intelligent Color Enhancement (FICE) or Blue Laser Imaging (BLI), Fujifilm, Japan) or indigo carmine chromoendoscopy was used. Indigo carmine chromoendoscopy was also routinely used in the evaluation of patients with FAP. Submucosal lifting was performed with saline stained with indigo carmine, with or without epinephrine according to the operator’s preference, a commercially available submucosal lifting solution (Orise, Boston Scientific, USA), or a 5% fructose and 10% glycerol submucosal lifting solution produced by the hospital pharmacy^8^.

Resection was performed with braided or condensed strand multifilament or single stranded (monofilament) resection snares. Diameters of the snares were 10 mm (SD-990– 10, Olympus), 15 mm (SD-990-15, Olympus), 20 mm (POL1-B7-20-23-220-OL, Medwork, Germany), 22 mm (POL1-B8-22-23-220-OL, Medwork,), 25 mm (SD-990– 25, Olympus) or 30 mm (POL1-B3-30-23-220-OL, Medwork). The resection was performed with the Q endocut mode on a VIO300 D or VIO 3 electrosurgical generator (Erbe, Tubingen, Germany). Through the scope clips (Resolution, Boston Scientific; Instinct, Cook medical; Novaclip, Vytil) were used for hemostasis, to close the mucosal defect whenever technically possible, or to treat immediate and delayed perforation.

Following the ESGE recommendations, antiplatelets were stopped 5 days before the procedure; anti-vitamin K drugs were stopped 5 days before the procedure until a maximum INR of 1.5 was reached; direct oral anticoagulants (Dabigatran, Rivaroxaban, Apixaban) were stopped 48–72 h before the procedure^5^. All patients were admitted to the hospital following the endoscopic procedure. Re-feeding was allowed the same evening unless otherwise instructed by the operator, and blood counts were monitored at day 1 and day 2 before discharge. A follow-up esophagogastroduodenoscopy (EGD) was planned at 3 months and at 1 year, or every 3 to 6 months in case of a local recurrence at follow-up EGD^5^.

### Outcomes

The primary outcome was the rate and the risk factors of clinically significant AE: delayed bleeding, perforations during the procedure, and delayed perforations. The secondary outcomes were the rate of local recurrence after endoscopic resection, defined as the occurrence of adenomatous lesion during follow-up endoscopy after complete endoscopic resection, the feasibility of the endoscopic treatment for recurrences, the number of endoscopic treatment sessions required, and the rate of surgical intervention.

### Adverse events:

AE were recorded during the procedure, during the 48 h of hospital admission following the procedure, and during a follow-up consultation one to three months after the endoscopy. The severity of the AE was assessed using the American Society of Gastrointestinal Endoscopy classification^9^. Delayed bleeding was defined as the presence of hematemesis and/or melena after the patient left the endoscopy room. Bleeding was classified as major when it resulted in a hemoglobin drop of more than 2 g/dL. Intraprocedural perforation was defined as a visible defect in the muscularis propria, associated or not with the visualization of periduodenal structures. Delayed perforation was diagnosed when post-procedural imaging justified by abdominal pain or general symptoms (fever, malaise, tachycardia) showed fluid in the periduodenal area or oral contrast extravasation.

### Statistical analysis:

Data was collected in a database using the Excel software (Microsoft Corp., Seattle, Washington, U.S.A), and analyzed with the Graphpad software (Graphpad, San Diego, CA) and Pvalue.io software (Pvalue.io, Medistica, Paris, France). Continuous variables are presented as mean ± standard deviation (SD) or median with ranges or interquartile range (IQR). Categorical variables are expressed as percentages. Comparison of numerical variables was performed using a Student test. A p value < 0.05 is considered significant. For the comparison of categorical data, a Fisher’s exact test was used. Univariate and multivariate analyses were used to determine independent risk factor of complications and recurrence. We included in multivariate analysis parameters with a positive association in univariate analysis and the use of antiplatelet or anticoagulant which is known to impact adverse events rates. The datasets used and/or analyzed during the current study available from the corresponding author on reasonable request.

### Ethical aspects

All patients signed a written consent for therapeutic endoscopy after being informed about the procedure, its benefits and risks, especially in terms of complications. All patients provided informed consent for the use of their medical data for medical research. The study was approved by our local ethics committee (Comité Local d’Ethique des Publications de l’Hôpital Cochin), CLEP -n° AAA-2021–08,014.

## Results

### Patients and lesions characteristics

**Figure 1:**
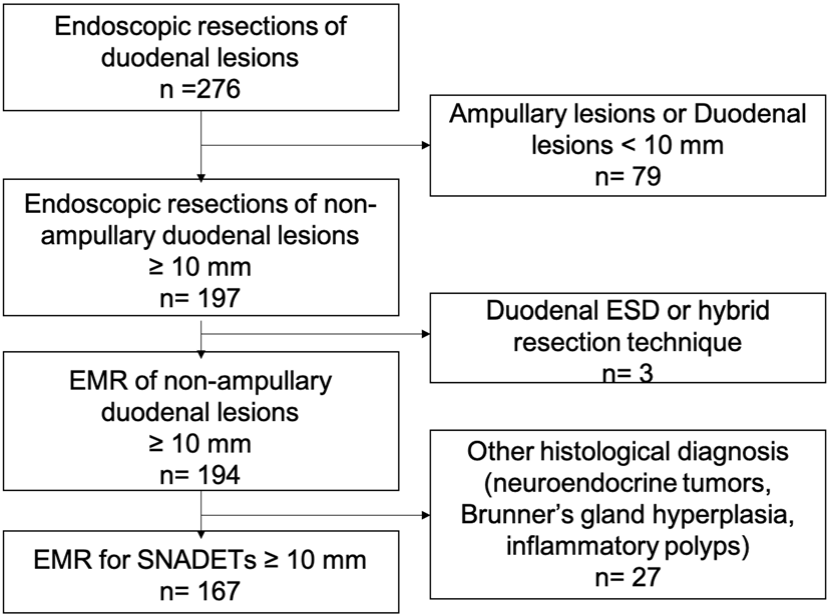
Study flowchart.

Between January 2015 and December 2020, 276 endoscopic resections were performed in 226 patients for duodenal lesions. Of these, 167 non ampullary duodenal adenomatous lesions of 10 mm and more were resected by EMR. We excluded infracentimetric lesions, submucosal dissection and other histological diagnosis. The study flowchart is presented in Fig. 1.

Patients and lesions’ characteristics are summarized in Tables 1 and 2. A total of 167 patients were treated for a SNADET with a median (IQR) follow-up time of 13.9 (3.7–26.9) months. The mean (± standard deviation) age was 62.2 (± 14.2) years and 90/167 (53.9%) were women. Some 17/167 (10.2%) patients were treated by antiplatelet and 11/167 (6.6%) by anticoagulant therapy. Sporadic lesions represented 118/167 (70.7%) of lesions, while 49/167 (29.3%) lesions were related to genetic predisposition syndromes, including 34/167 (20.4%) patients with FAP. The vast majority of duodenal adenomatous lesions were located in the second part of the duodenum (133/167, 79.6%). Paris 0–IIa subtype was recorded in 149/167 (89.2%) cases, and the median (IQR) size was 25 (25–40) mm. 94/167 (56.3%) lesions were considered as large (< 30 mm) and 73/167 (43.7%) as giant (≥ 30 mm). Low-grade dysplasia was recorded in 64/167 (38.3%), high-grade dysplasia in 102/167 (61.1%), and invasive carcinoma in 1/167 (0.06%) of the SNADETs.

**Table 1:** Baseline patients characteristics.

Patients—n	n = 167
Age (mean ± SD), years	62.2 ± 14.2
Sex, Male/Female—n	77/90
ASA score 1/2/3—n	77/57/33
Antiplatelet agent—n (%)	17 (10.2)
Anticoagulation (VKA/DOA)—n (%)	11 (6.6)
Hereditary predisposition syndrome—n (%)	49 (29.3)
FAP	34 (20.4)
Lynch syndrome	2 (1.2)
MUTYH polyposis	6 (3.6)
Juvenile polyposis	1 (0.6)
Peutz-Jeghers syndrome	3 (1.8)
**Sporadic**—n (%)	118 (70.7)
**Concurrent colonic adenoma/Colonoscopy—n (%)**	83/106 (78.3%)
Hereditary predisposition syndrome	44/48 (91.7%)
Sporadic	39/58 (67.2%)

VKA: Vitamin K antagonist; DOA: Direct oral anticoagulant; n: number; SD: standard deviation; ASA: American society of anesthesiologists; FAP: Familial adenomatous polyposis; D1: First duodenum; D2 Second duodenum.

**Table 2:** Baseline lesions characteristics.

Lesion site—n (%)	
D1	17 (10.2)
D2	133 (79.6)
D3/D4	17 (10.2)
**Size in mm, median (IQR)**	25 (25–40)
**Size repartition—n (%)**	
10–29 mm	94 (56.3)
≥ 30 mm	73 (43.7)
**Paris Classification—n (%)**	
0–Is	16 (9.6)
0–IIa	149 (89.2)
0–Ip	2 (1.2)
**Histology—n (%)**	
Low-grade dysplasia	64 (38.3)
High-grade dysplasia	102 (61.1)
Invasive adenocarcinoma	1 (0.06)
Time of follow up in month—median (IQR)	13.9 (3.7–26.9)

D1: First duodenum; D2 Second duodenum; D3/4: third/fourth duodenum; n: number; mm: millimeter; IQR: Interquartile range.

### Endoscopic characteristics

The endoscopic characteristics of the SNADETs are summarized in Table 3. EMR was performed with a gastroscope in 103/167 (61.7%) cases and with a duodenoscope and a pediatric colonoscope respectively in 21/167 (12.6%) and 43/167 (25.7%) cases. A distal attachment cap was added in 43/124 (34.7%) endoscopies. EMR were typically performed with a 15 mm monofilament endoscopic resection snare (98/167, 58.7%), after submucosal lifting with a mixture of saline and indigo carmine (127/167, 76.0%). En bloc resection was achieved in 63/167 (37.7%) cases. The resection bed was closed with clips in 65/167 (38.9%) of cases.

**Table 3:** Endoscopic procedural characteristics.

**Type of endoscope—n (%)**	
Gastroscope- n (%)	103 (61.7)
Duodenoscope—n (%)	21 (12.6)
Pediatric Colonoscope—n (%)	43 (25.7)
Cap—n (%)	43 (25.7)
**Submucosal injection—n (%)**	
Saline and indigo carmine	127 (76.0)
Glycerol-Fructose	37 (22.2)
Commercial submucosal lifting gel	3 (1.8)
**Endoscopic resection snare type -, n (%)**	
Braided or condensed multifilament snare	79 (47.3)
Monofilament snare	88 (52.7)
**Snare diameter—n (%)**	
10 mm	30 (18.0)
15 mm	98 (58.7)
20 mm	21 (12.6)
25 mm	12 (7.2)
30 mm	6 (3.6)
**Resection modality—n (%)**	
En bloc	63 (37.7)
Piecemeal	104 (62.3)
**Closing by clips—n (%)**	65 (38.9)
**Length of hospital stay, median (IQR), days**	2 (2–2)
**Adverse events—n (%)**	37 (22.2)
Delayed bleeding	29 (17.7)
Immediate perforation	4 (2.4)
Delayed perforation	4 (2.4)

### Adverse events

AE occurred in 37/167 (22.2%) resections with 29/167 (17.7%) delayed bleedings, 4/167 (2.4%) immediate perforations and 4/167 (2.4%) delayed perforations. Univariate analysis showed a positive association with AE and giant vs. large duodenal adenomatous lesions (34.2% vs 12.8%, p < 0.001), monofilament snares vs. braided or condensed strands snares (28.4% vs 15.2%, p = 0.04), and not using clips (29.3% vs 15.3%, p = 0.003) (Table 4). In multivariate analysis, only giant lesions were associated with AE (OR = 2.81, 95% CI [1.27; 6.47], p = 0.012) (Table 5). There was no association between the occurrence of clinically relevant AE and patients’ or lesions’ characteristics: age, sex, medical history, sporadic or genetic syndrome, the use of antiplatelet agent or anticoagulant, localization of the lesion in the duodenum; nor with resection techniques: type or size of the resection snare, closure with clips, type of endoscope, presence of a cap, type of submucosal lifting solution, adjunction of epinephrine.

**Table 4:** Adverse events after endoscopic mucosal resection for duodenal adenomas in univariate analysis.

		No AE (n = 130)	AE (n = 37)		n	p
Numbers of clips	0	58 (70.7%)	24 (29.3%)		82	0.003
	≥ 1	72 (85.7%)	13 (15.3%)		85	
Size, in mm	< 30 mm	82 (87.2%)	12 (12.7%)		94	< 0.001
	> 30 mm	48 (65.8%)	25 (34.2%)		73	
Resection snare	Braided or condensed multifilament snare	67 (84.8%)	12 (15.2%)		79	0.04
	Monofilament snare	63 (71.6%)	25 (28.4%)		88	
Age (mean ± SD)		62.0 (14.4%)	62.9 (13.6%)		167	0.72
ASA Score	0	2 (2%)	1 (3%)		3	0.63
	1	60 (46%)	14 (38%)		74	
	2	44 (34%)	13 (35%)		57	
	3	24 (18%)	9 (24%)		33	
Antiplatelet/Anticoagulant	No	103 (79%)	33 (89%)		136	0.17
	Yes	27 (21%)	4 (11%)		31	
Adjunction of epinephrine	No	110 (85%)	33 (89%)		143	0.87
	Yes	20 (15%)	4 (11%)		24	
Bed closing with hemoclips	No	75 (58%)	27 (73%)		102	0.09
	Yes	55 (42%)	10 (27%)		65	
Cap	No	98 (75%)	26 (70%)		124	0.53
	Yes	32 (25%)	11 (30%)		43	
Type of endoscope	Gastroscope	83 (64%)	20 (54%)		103	0.19
	Pediatric colonoscope	13 (10%)	8 (22%)		21	
	Duodenoscope	34 (26%)	9 (24%)		43	
FAP	No	104 (80%°	29 (78%)		133	0.83
	Yes	26 (20%)	8 (22%)		34	
Localization	D1	14 (11%)	3 (8.1%)		17	0.84
	D2	102 (78%)	31 (83.8%)		133	
	D3/4	14 (11%)	3 (8.1%)		17	
Resection	En bloc	54 (42%)	9 (24%)		63	0.057
	Piecemeal	76 (58%)	28 (76%)		104	
Sex	Male	58 (45%)	19 (51%)		77	0.47
	Female	72 (55%)	18 (49%)		90	
Submucosal lifting solution	Saline solution	98 (75%)	29 (78%)		127	0.14
	Glycerol fructose	31 (24%)	6 (16%)		37	
	Lifting gel	1 (1%)	2 (6%)		3	

AE: adverse events, n: number; D1: first duodenum; D2: Second duodenum; D3/4: third & fourth duodenum; FAP: familial adenomatous polyposis.

**Table 5:** Adverse events after endoscopic mucosal resection for duodenal adenomas in multivariate analysis.

		Odds-Ratio	p
Size, in mm	≥ 30 vs 10–29 mm	2.81 [1.27; 6.47]	**0.012**
Resection snare	Mono vs. multifilament	2.03 [0.92; 4.66]	0.084
Numbers of clips used	0 vs. ≥ 1	0.53 [0.23; 1.19]	0.11
Antiplatelet/anticoagulant	No vs. Yes	0.4 [0.11; 1.18]	0.13

A lesion size > 30 mm (26% vs. 10.6%, p < 0.01), a type 0–Is of the Paris classification (41.2% for 0–IIa lesions; 14.7% for 0–Is lesions; 0% for 0–Ip lesions, p = 0.044) and the use of monofilament snare (23% vs. 11.3% for multifilament snare, p = 0.045) were significantly associated with a greater occurrence of delayed bleeding.

### Management of adverse events after endoscopic resection

Delayed bleeding was managed during a second endoscopy by clipping the bleeding site in 16/29 (55.2%) patients, by applying hemostatic compounds, such as Purastat (3D Matrix, Japan) or Hemospray (Cook Medical, USA) in 5/29 (17.2%) patients, or by conservative medical treatment in 8/29 (27.5%) patients. Thirteen (44.8%) patients had red blood cell transfusions with median (IQR) of 2 (2–5) red blood cell.

In case of immediate perforation, through the scope clips were used in 2/4 (50%) patients to close the defect, an over the scope clip was used in 1/4 (25%) patient and surgery was necessary in 1/4 (25%) patient.

Delayed perforation was managed surgically in 2/4 (50%) patients, or by placing through the scope clips in 1/4 (25%) patient or an over the scope clip in 1/4 (25%) patient.

The median (IQR) hospital stay was significantly longer in patients experiencing AE (4 (2–8), p < 0.001), delayed bleeding (4 (2–7), p < 0.01), immediate perforation (9.5 (6–16), p < 0.01) and delayed perforation (18 (15–21), p < 001) vs no AE (2 (2–2)). No patient died following duodenal EMR.

### Recurrence

For the analysis of recurrence, we included all patients with at least one follow-up endoscopy without a hereditary predisposition syndrome. A total of 80 patients were included. Local recurrence occurred after 34/80 (42.5%) endoscopic resections of centimetric or supracentimetric duodenal adenomatous lesions. Univariate analysis showed that a high-grade dysplasia (51.2% vs. 15%, p < 0.001), a piecemeal resection (52.1% vs. 28.1%, p = 0.034), and multiple resected lesions (80% vs. 37.1%, p = 0.015) were potential risk factors for local recurrence (Table 6). The first endoscopic follow-up was normal in 60/80 (66.7%) patients. Among patients with recurrence, there were a median (IQR) of 2 (3–1) endoscopic resections. 2/80 (2.5%) patients underwent surgery after endoscopic treatment failure.

**Table 6:** Factors influencing local recurrence after endoscopic mucosal resection for duodenal adenoma, univariate analysis.

		No Recurrence (n = 46)	Recurrence (n = 34)	n	p
Histology	LGD	17 (37%)	3 (8.8%)	20	< 0.01
	HGD	29 (63%)	31 (91.2%)	60	
Multiple resected lesions	Yes (> 1)	44 (96%)	26 (76%)	70	0.015
	No (≤ 1)	2 (4%)	8 (24%)	10	
Resection	En-bloc	23 (50%)	9 (26%)	32	0.034
	Piecemeal	23 (50%)	25 (74%)	48	

LGD: Low-grade dysplasia; HGD: High-grade dysplasia.

## Discussion

Among 167 SNADETs over a period of 5 years, we recorded a 22.2% rate of clinically significant AE, and found a lesion size ≥ 30 mm to be the only statistically significant risk factor for AE. Noticeably, the risk of AE was not associated with patients’ or other lesions’ characteristics, and currently recommended technique prophylactic measures, such as the closure of the resection bed with clips, had no influence on the risk of AE. The use of a monofilament snare allows, due to an increased rigidity, to better grasp a flat lesion. However, the cutting phase is quicker, possibly resulting in less coagulation of the resection bed, and in a higher secondary bleeding rate explaining the trend although the difference was not statistically significant.

Retrospective studies suggested that delayed bleeding occurred in 4.4–17.4% of cases after EMR of duodenal adenomatous lesions^10–17^, and that the risk increased with the size of the lesion and the presence of a protruding (Paris type 0–Is) lesion. In the only currently published prospective study including 110 lesions and 118 patients, delayed bleeding occurred in 18.6% of cases^18^. Although this high rate can be the result of the inclusion of 18% ampullary adenomas, and 30% giant adenomas, it also underlines the possible underestimation of the complications rates after duodenal EMR in most retrospective studies. Aschmoneit-Messer et al., in a prospective study including 50 patients and 61 lesions, showed that prophylactic argon plasma coagulation (APC) of the resection bed lowered the risk of delayed bleeding after EMR of duodenal adenomatous lesions > 20 mm and/or in case of visible vessels ≥ 1 mm^19^. However, considering a delayed perforation in one out of the six patients managed with APC, the safety of this attidude is debatable. Lepilliez et al., in a retrospective study including 36 patients and 37 lesions, found that no delayed bleeding occurred in patients treated by prophylactic clipping or prophylactic argon plasma coagulation, or in patients treated for intraprocedural bleeding. In the meantime, delayed bleeding occurred in 21.7% of the rest of the patients^16^. Nonaka et al., in a retrospective study including 113 patients and 121 lesions, showed that delayed bleeding rate dropped from 32 to 7% in cases of prophylactic clipping (p < 0.004)^17^. These two works, along with others, have contributed to demonstrate that complete closure by clips, feasible in most of the cases for lesions of less than 20 mm, is effective in preventing adverse events^20^. However, in the large lesions included in our study, complete closure was only feasible in 38.9% of the lesions. Therefore, we cannot exclude that a complete closure of these large lesions would have resulted in a reduced adverse event rate. Therefore, ESGE guidelines recommend prophylactic treatment of delayed bleeding by placing clips to close the mucosal defect or by non-contact hemostatic measures^5^. Considering the questionable stability and efficacy of hemostatic powders and gels, the most promising options for AE prevention after duodenal endoscopic resection rely in a change of the resection technique, using cold EMR, associated with a lower rate of AE; and improved mucosal defect closure, using endoscopic suturing techniques when complete closure by clips is impossible.

While the 17.7% delayed bleeding rate was in keeping with the literature data, we did not observe any statistically significant benefit of clipping the resection bed. This is likely to be explained by the large size of the resected lesions, with over 45% of lesions > 30 mm, precluding a complete closure of the resection bed with clips.

Immediate perforation, defined by a breach in the muscularis propria during endoscopic resection, occurs in 2.2–6% of the resections^10–17^. The management of immediate perforations consists in the closure of the perforation with clips, preferably after completing the resection. Perforation typically occurs in pretreated or multibiopsied lesions with submucosal fibrosis, or insufficient submucosal injection. In our cohort, immediate perforation occurred in 2.4% of the resections.

Delayed perforations of the duodenal wall are likely caused by multiples parameters such as thermic aggression from the resection and hemostasis, but also chemical aggression by bile acids and pancreatic fluid. After EMR for duodenal adenomatous lesions, the reported rates range from 1.7 to 7.4%, and account for the 1% mortality associated with this procedure^10–17^. Our findings were in line with these numbers, with no mortality, and also illustrate the feasibility of endoscopic management of delayed perforations.

Based on retrospective studies, local recurrence rate ranges from 9 to 37%^10,13–15^. It appears to be maximal for piecemeal resections of lesions > 20 mm in size. We found an overall 42.5% recurrence rate and identified high-grade dysplasia, piecemeal resection and multiple resected lesions as potential risk factors. As recurrence was a secondary endpoint we did not perform a multivariable analysis. These high numbers can be explained by the high proportion of piecemeal resection due to the large size of the lesions resected in our cohort.

The strengths of our study were the large number of resections, performed in consecutive and prospectively recorded patients at a single center, including large and giant lesions, with available follow-up data, allowing to assess the recurrence rates. Main limitations are the heterogeneity of the resection tools, reflecting the number of operators involved, the retrospective analysis of the database, leading to loss of data, and the inclusion of patients from an expert center, leading to a selection bias. In addition, multivariable analysis was performed only for the main outcome and not for the secondary endpoints. As the information regarding the location relative to the papilla was most often missing, we could not analyze this parameter.

Underwater EMR (U-EMR) performed in the duodenum is a promising treatment option. Filling the duodenal lumen with water prevents the excessive stretching of the duodenal wall and allows to resect duodenal neoplasms without submucosal lifting. These two factors allowed to reach 87.5–100% complete endoscopic resection with a delayed bleeding rate reaching 25%, and low perforation rate^21–25^. Endoscopic submucosal dissection allows en-bloc resection rates of 75–100%, with 9–36% of perforations. In addition, duodenal ESD does not result in a lower rate of local recurrences^26^. As shown in multiples studies, cold snare polypectomy appears to be a safe resection technic for duodenal lesions up to 10 mm^27,28^. Given these figures, ESGE recommends cold snare polypectomy for lesions up to 6 mm and to limit the use of duodenal endoscopic submucosal dissection to selected cases in expert centers^12,14,16,29,30^.

Future measures are still at an experimental stage in 2022. Endoscopic suturing of duodenal EMR sites has been described as feasible in a case series of 7 patients^31^. Considering the size, cost, and maneuverability of the available endoscopic suturing devices, endoscopic suturing of EMR sites is not cost-effective. Upper gastrointestinal full thickness resection device has been performed in 8 patients with duodenal lesions^32^ with an excellent safety profile, but technical feasibility and histologically complete resection rates in lesions > 20 mm is uncertain.

In conclusion, EMR for supracentimetric duodenal adenomatous lesions is associated with AE such as delayed bleeding or delayed perforation in 22.2% of the cases, particularly in lesions ≥ 30 mm. Preventive measures, such as the complete closure of the mucosal defect with clips is often technically impossible in large lesions, while prophylactic coagulation of the resection bed might increase the risk of delayed perforation.
